# Nullomer peptide increases immune cell infiltration and reduces tumor metabolism in triple negative breast cancer mouse model

**DOI:** 10.21203/rs.3.rs-3097552/v1

**Published:** 2023-06-28

**Authors:** Nilufar Ali, Cody Wolf, Swarna Kanchan, Shivakumar R. Veerabhadraiah, Laura Bond, Matthew W. Turner, Cheryl L. Jorcyk, Greg Hampikian

**Affiliations:** Boise State University; Boise State University; Boise State University; University of Utah; Boise State University; Boise State University; Boise State University; Boise State University

**Keywords:** breast cancer, triple negative breast cancer, TNBC, nullomer, peptide, tumor microenvironment

## Abstract

**Background:**

Nullomers are the shortest strings of absent amino acid (aa) sequences in a species or group of species. Primes are those nullomers that have not been detected in the genome of any species. 9S1R is a 5-aa peptide derived from a prime sequence that is tagged with 5 arginine aa, used to treat triple negative breast cancer (TNBC) in an in vivo TNBC mouse model. 9S1R is administered in trehalose (9S1R-NulloPT), which enhances solubility and exhibits some independent effects against tumor growth and is thus an important component in the drug preparation.

**Method:**

We examined the effect of 9S1R-NulloPT on tumor growth, metabolism, metastatic burden, necrosis, tumor immune microenvironment, and the transcriptome of aggressive mouse TNBC tumors.

**Results:**

The peptide-treated mice had smaller tumors in the initial phase of the treatment, as compared to the untreated control, and reduced in vivo bioluminescence at later stages, which is indicative of metabolically inactive tumors. A decrease in ex vivo bioluminescence was also observed in the excised tumors of treated mice, but not in the secondary metastasis in the lungs. The treatment also caused changes in tumor immune microenvironment with increased infiltration of immune cells and margin inflammation. The treatment upregulated 365 genes and downregulated 710 genes in tumors compared to the untreated group. Consistent with in vitro findings in breast cancer cell lines, downregulated genes in the treated TNBC tumors include Cellular Metabolic Process Related genes (179), specifically mitochondrial genes associated with TCA cycle/oxidative phosphorylation (44), and translation machinery/ribosome biogenesis genes (45). Among upregulated genes, the Developmental Pathway (13), ECM Organization (12) and Focal Adhesion Related Pathways (7) were noteworthy. We also present data from a pilot study using a bilateral BC mouse model, which supports our findings.

**Conclusion:**

In conclusion, although 9S1R-NulloPT was moderate at reducing the tumor volume, it altered the tumor immune microenvironment as well as the tumor transcriptome, rendering tumors metabolically less active by downregulating the mitochondrial function and ribosome biogenesis. This corroborates previously published in vitro findings.

## Background

The chance of a woman being diagnosed with breast cancer (BC) during her lifetime is 1 in 8 [1]. In the US, invasive breast cancer is predicted to be diagnosed in an estimated 297,790 women and 2,800 men in 2023 [2] and by 2040, with no major changes in prevention or treatment, 1.4 million women will die from BC worldwide [3]. There is a well-established heterogeneity in BC subtypes with presence, absence or a combination of estrogen receptor alpha (ER ), human epidermal growth factor receptor (HER) and progesterone receptors (PR), that dictate treatment strategies. Based on SEER (Surveillance, Epidemiology, and End Results) [4] data in the United States 12% of breast tumors are triple-negative, which lack ER-, PR- and HER2- (triple-negative BC -TNBC) [5]. This subtype is the most aggressive, with the highest level of invasiveness and worst prognosis, resulting in 25–46% of brain metastasis [4]. BC treatment consists principally of surgery, radiation therapy, chemotherapy, hormonal therapy, targeted antibody or small-molecule therapy. Ground breaking approaches such as CAR-T cell therapy show only modest effects on solid tumors [6] and few drugs have been approved by the U.S. FDA for patients with TNBC tumors. PD-1 (programmed cell death protein-1) inhibitor in combination with chemotherapy is one such drug but is limited to patients with tumors expressing PD-L1 (programmed cell death-ligand 1), and those with high-risk, early stage TNBC [7]. The only approved targeted therapy for TNBC is a Trop-2 targeted antibody-and topoisomerase I inhibitor conjugate, used as a second-line treatment for unresectable locally advanced or metastatic triple-negative breast cancer (mTNBC) [8, 9]. Recent cancer immunotherapy drug development is focused on immunological agents that augment the natural immune response of patients against cancer-specific antigens [10]. While therapies involving adjuvant immunotherapies, oncolytic viruses, cytokines, antibodies, peptides, and their combinations are in clinical trials for BC treatment, there are certain drawbacks associated with each such as limited efficacy, lack of response, higher cost and poor accessibility for lower income nations. Peptide based drugs are showing great promise in clinical studies [11–13]. Peptides offer the advantage of small size, specificity, effects on a broad range of cancers, low toxicity and low manufacturing cost [13]. In the current study, we investigated the therapeutic potential of a novel 10 amino acid nullomer-peptide (NulloP) in a TNBC mouse model.

Nullomers are the shortest absent sequences in a species (or group of species). These can be nucleotide strings, or amino acid strings (Nullopeps, or NulloPs). The set of nullomers absent from the entire biome (as represented in available databases) are called primes (DNA) or peptoprimes (amino acids) [14–16]. Both Nullomers and primes are seeds from which other absent sequences can be constructed by adding to either end of the string. The nullomer approach to drug design is based on a simple proposition that the smallest absent sequences can affect reactions when injected into hosts. There are two main hypotheses regarding the effects of injected nullomers on cancer cells, they may be toxic or immune stimulatory. The toxic hypothesis is based on the idea that evolution is the longest running experiment in biology, and selection acts to restrict or amplify the permutations of nucleotide and amino acid sequences that arise by chance. In the most extreme case, sequences that conflict with the basic common elements of cell physiology (ribosomes, mitochondria etc.) may be completely “forbidden” [14, 17]. The immunogenic hypothesis is based on the idea that the immune system identifies short sequences in a polymer as self or non-self. We could view the immune system as a nullomer search and destroy mission, thus nullomer-based drugs may increase immune responses. The original peptoprime sequences described in 2007 [14] are five amino acids long and have been modified with the addition of five arginine molecules to enhance cell-penetrating properties [17, 18]. When dissolved in trehalose, several of the peptoprimes (NulloPTs) were shown to be preferentially lethal to breast and prostate cancer cells, as opposed to normal primary cell lines [17–19]. Trehalose is reported to have an independent effect against BC [20–24] and is an important component in our drug preparation. Nullomers have also been used to design vaccines from epitopes present in the host but absent in a pathogen and to make DNA “watermarks” for labeling by combining them in series [19].

The original 198 peptoprimes (and scrambled versions of their sequences) were assayed in vitro against the NCI 60 cancer lines [18], and 9S1R, a potent killer of cancer cells was selected for the mouse experiments reported here. 9S1R is a scrambled version of an original peptoprime, that is a nullomer (absent) from the biome. 9S1R-NulloPT is absent from the human and mouse proteomes and has been shown to dramatically decrease ATP production, inhibit mitochondrial FoF_1_-ATP synthase, reduce mitochondrial membrane potential and increase superoxide free radicals inside the mitochondria of MDA-MB-231 human TNBC cells and the NCI 60 cancer cell panel, ultimately killing them [17, 18]

This is the first in vivo study of nullomer-based peptides. Here we report the therapeutic effects of 9S1R-NulloPT in the 4T1.2-Luc mouse TNBC model. The 9S1R-NulloPT was initially tested in a bilateral tumor model **(pilot study-1, details in Supplementary)** with six administrations at two different doses (50mg/kg and 100mg/kg). Following these results, we moved to a unilateral tumor model with eight drug administrations at the most effective dose (100mg/kg). Although our preclinical in vitro studies showed a substantial response against diverse panels of cancer cells, the present in vivo study in mice showed that the drug effectively reduces TNBC tumor size at the initial growing phase of the tumor, but not in the advanced stage. 9S1R-NulloPT changes the tumor immune microenvironment and affects tumor energy metabolism specifically targeting mitochondrial and ribosomal genes, providing a segue for future studies targeting tumor mitochondria and ribosome synthesis in TNBC.

## Methods

### 1. Drug preparation-

9S1R nullomer peptide (sequence RRRRRWCMNW) was synthesized by Genscript (USA) (lyophilyzed, HPLC purified, purity > 98%) and stored at − 20°C. For preparation of 9S1R-NulloPT, briefly, 20 μg/μl stock solution of the 9S1R peptide was prepared in 100mM Trehalose (Sigma-Aldrich, USA), which was then used as mg/kg body weight for injections in mice. For example, a mouse of 25g body weight for dose 100mg/kg, received 200ul of drug formulation containing 100ug/g bodyweight of 9S1R peptide mixed with 2.5mM/g bodyweight of Trehalose prepared in PBS. Trehalose only group received 200ul of 2.5mM trehalose in PBS per gram bodyweight, and PBS group received 200ul of PBS. All drug preparations were filtered through 0.22μm filter (Millipore, USA) before IP administration.

### 2. Single-dose acute toxicity study-

A single dosage of the 9S1R-NulloPT drug at 5, 25, 50, 100 mg/Kg and highest equivalent dose of Trehalose alone (2.5mM/g bodyweight) was injected in mice (n = 4) intraperitoneally (IP). Mice were observed every 2h for clinical signs and symptoms and body weights were taken every 12h. At the end of the experiment on 36h mice were euthanized, necropsy was performed and organs preserved in 10% formalin solution.

### 3. Cell culture:

Mouse triple-negative 4T1.2 and 4T1.2-Luc cells were cultured and maintained as previously described [25] with α-MEM supplemented with 10% fetal clone III serum (Cytiva, USA), 1% penicillin/streptomycin and 1mM sodium pyruvate. Cells were maintained at 37 C, 5% carbon dioxide, and 100% humidity in a sterile tissue culture incubator.

### 4. The TNBC mouse model and treatment paradigm-

The model was created by orthotopically injecting 4T1.2-Luc cells in the 4th mammary fat pad of Balb/c mice, with 1×10^5^ cells per mouse. The cell line was developed by Dr. Cheryl Jorcyk [25] and it expresses luciferase gene which serves as a luminescent indicator of gene expression or tumorigenesis, making the tumor cell traceable in vivo. Treatment started after the formation of palpable tumors and was detected by in vivo BLI (IVIS, Perkin Elmer) followed by randomization of the animals per group. For evaluating the effect of the peptides on this model, we carried out two separate studies. The first pilot study involved a set of 4 mice per group with bilateral mammary tumors, which received six IP administration of PBS, 9S1R-NulloPT drug at 50 mg/kg, 100 mg/Kg body weight or Trehalose, over a period of 2 weeks. The present study includes a set of 7–9 mice per group with unilateral tumors including the same groups as previous, but received a total of eight injections per mouse over a period of 2 weeks. The animals were euthanized after 29 days post-tumor cell transplantation, the details are provided in the Supplementary section. The group which received IP injections of 9S1R-NulloPT drug at 100 mg/kg dose is regarded as the treated group, whereas the control group is PBS, unless mentioned otherwise.

### 5. Treatment groups-

The 9S1R peptide at dose 100mg/kg in trehalose is referred to as 9S1R-NulloPT or the treatment group throughout this paper. PBS was used as the control. Results from all groups (PBS, 9S1R-NulloPT at dose 50 mg/kg, 9S1R-NulloPT at 100 mg/Kg, and trehalose) are provided in the supplementary section.

### 6. Tumor volume measurement-

Mice were anesthetized by isoflurane followed by measurement of the tumor length and width using a manual Vernier caliper. This was performed before every drug administration and the tumor volume was calculated using the formula (length × width^2^)/2. Body weights were also measured before every dose of the drug.

### 7. In vivo imaging and Luciferase reporter-

Whole body bioluminescence imaging (BLI) by IVIS^®^ Spectrum in vivo imaging system (Perkin Elmer) was performed to detect in vivo tumor burden and metastasis, as evaluated by the bioluminescence signal from the 4T1.2-Luc cells. For confirmation ex vivo BLI was performed from the excised tumor, other organs and secondary metastasis sites. Mice were injected with 200 μl of D-luciferin (150 mg/kg) once before usual in vivo BLI and reinjected once more at the endpoint before necropsy and ex vivo BLI. The Images were analyzed by Perkin Elmer software and Aura Version 4.0.7 (Spectral instruments Imaging)

### 8. Histology-

Half of the tumor tissue was excised and sent for histopathology analysis by a practicing pathologist at the COBRE-Histopathology Imaging Core at Boise Veterans Affairs Research Department. The tissues were fixed, paraffin-embedded, and sectioned (1μm) followed by H&E staining. The slides analyzed for tumor grade, stage, necrosis, aggressiveness, margin inflammation and immune cell infiltration. Representative images from the H&E stained slides were captured at brightfield setting of microscopes ECHO Revolve (Bico, USA) and EVOS M50000 (Invitrogen, USA). Images were captured close to the edge of tumor border and stroma of all tumors.

### 9. Half-life of peptide 9S1R-

The half-life of peptide 9S1R was determined by spiking the peptide into fetal bovine serum (FBS) for exposure times of 30 seconds, 5, 30, 60 or 90 min. During the exposure, FBS containing the spike peptides were incubated at 37°C. The exposure was halted, and the peptide was extracted from FBS using protein precipitation. Protein precipitation was accomplished using 75% ice cold acetonitrile, followed by incubation at −20°C for one hour, and centrifugation at 9,000 rpm for 10 min at −4°C. The supernatant was removed and saved for analysis. Peptide samples were analyzed by High pressure liquid chromatography (HPLC) mass spectrometry (MS) using an ultra-high resolution Quadrupole Time of Flight (QTOF) instrument (Bruker maxis, Bruker Corporation, Billerica, MA, USA). HPLC mobile phase consisted of 18 MΩ H2O and HPLC grade formic acid and acetonitrile (> 99% purity, Fisher Scientific, Pittsburgh, PA, USA). The electrospray ionization (ESI) source was operated under the following conditions: positive ion mode; nebulizer pressure: 1.2 Bar; flow rate of drying gas (N2): 8 L/min; drying gas temperature: 200°C; voltage between HV capillary and HV end-plate offset: 3000 V to − 500 V; mass range was set from 250 to 2900 m/z; and the quadrupole ion energy was 4.0 eV. Low concentration ESI tuning mix (Agilent Technologies, Santa Clara, CA, USA) was used to calibrate the system in the mass range. HPLC separation was achieved using a Dionex UltiMate^®^ 3000 RSLCnano system (Dionex Corporation, Sunnyvale, CA, USA) equipped with a Waters XTerra C18 column (4.6 × 100 mm, 3.5 μm) (Waters Corporation, Milford, MA, USA). The mobile phase was 0.1% formic acid in water (Buffer A) and acetonitrile (Buffer B) with a flow rate of 0.2 mL/min. A linear gradient method was used to separate the mixture starting at 5% acetonitrile and ending at 60% acetonitrile over 20 minutes. The sample injection volume was 5 μL. Data were analyzed using the Compass Data Analysis software package (Bruker Corporation, Billerica, MA, USA).

### 10. RNA sequencing-

At termination, tumors (n = 3) were excised, snap-frozen and sent to Novogene for RNA. Paired end raw sequences (sequenced using Illumina platform (PE150) were subjected to quality check using FastQC v0.11.9. Sequence reads for all genes in all samples were mapped to the Genome Reference Consortium Mouse build 38 (GRCm38) release 99 by HISAT2 v2.1.0 using Ensemble ID. Aligned sequence reads for each gene for all samples were assessed by HTSeq v0.11.3 using the GRCm38 (release 99) ensembl reference genome annotation (gtf format) file. Genes with greater than 10 sequence read counts (for each row considering 3 replicates in a sample) per gene in each sample were used for further analysis. Differential gene expression for each comparison (3 replicates for each sample) was performed by default Wald test using Deseq2 v1.38.1 in R. Normalization of sequence reads per gene count for each sample was done using median-ratio-normalization. Volcano plots, PCA plots, heatmap of samples (using Euclidean distances), heatmap of expression of top variable genes were generated for using gplots, ggplot2 and RColorBrewer programs (R based). Fold-change for each gene was calculated by comparing counts in all the treatments relative to vehicles and PBS. Genes with Benjamini-Hochberg (BH) adjusted p-value < 0.05 were considered differentially expressed genes (DEGs). Differentially expressed genes having log2 fold change value of at least 0.58 or greater were used for investigating significant biological processes, molecular functions, cellular compartments and biological pathways using gProfiler web server.

### 11. Interactome and cluster analysis-

To explore if the DEGs are involved in known and predicted protein-protein interactions, the TNBC-related PPI network was constructed using the online analysis tool STRING (Ver 11.2). The network nodes were the downregulated and upregulated DEGs and network edges shown in confidence view without any disconnected nodes in the network and with active interaction sources from experiment, databases, co-expression, neighborhood, gene fusion and co-occurrence [26]. The network was clustered to 3 groups following kmeans clustering with hidden edges between clusters for a simplified view. The interaction score was > 0.4 [27].

### 12. Statistical Analysis-

Two-tailed unpaired Student’s t-test was used for experimental statistics with p < 0.05 considered as statistically significant. Students’ T test and one-way ANOVA followed by post hoc test. GraphPad Prism (ver 9.5.1) was used for analysis.

## Results

### Characterization of the peptide 9S1R

**Figure 1:**
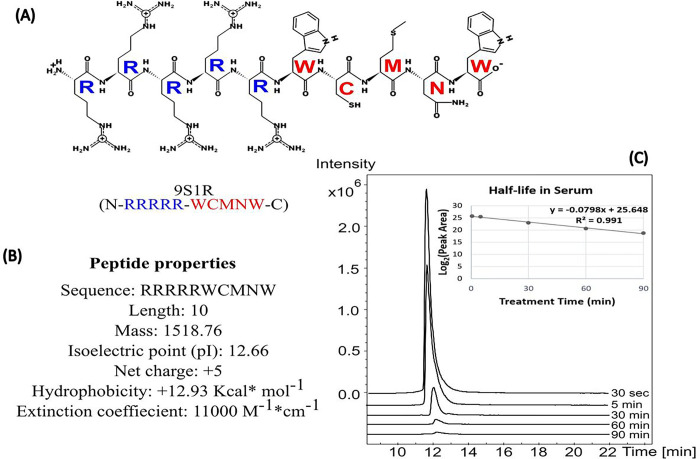
Characterization of 9S1R nullomer peptide. **(A)** Projected biochemical structure, blue represents poly-arginine and red is the 5 aa nullomer sequence (WCMNW). **(B)** physical properties of the peptide as obtained from Pepdraw (©2015). **(C)** The extracted ion chromatogram (EIC) peaks of the peptide after LC-MS at different incubation times in serum (0.5–90 min). The X-axis depicts the retention time and Y-axis peak intensity. Inset shows the half-life log_2_ peak area under the curve versus treatment time for the EIC peaks. t1/2 value for the peptide is 8.7 min.

9S1R (N-RRRRR-WCMNW-C) nullomer has a molecular weight 1519.81 Da and iso-electric point pH 12.28. It has a net charge of + 5 at pH 7, hydrophobicity + 12.93 Kcal/mol with amphipathic nature and fair solubility in water (Fig. 1A, B). We evaluated the uptake of the peptide inside 4T1.2 cells by mass spectrometry (MS) and found that the peptide is available inside the cells at two molecular forms with charges 3+ (m/z 507.2637 Da) and 4+ (m/z 380.7002 Da), (extracted ion chromatogram (EIC) peaks, **Supplementary Fig. 1**). We determined using MS that the half-life of the peptide in fetal bovine serum (FBS) was 8.7 mins (Fig. 1C). The previous in vitro study by Alileche et al. showed that this peptide targets mitochondrial function [18]. This is in agreement with the above properties of this molecule, in that it is very similar to known mitochondria-targeting peptides (MTPs) [28–31] and, we hypothesize that the peptide localizes to mitochondria as an MTP. Thus, we determined that the peptide enters cells and has a short half-life in serum.

### Single dose acute toxicity study of 9S1R NulloPT in mice.

**Figure 2:**
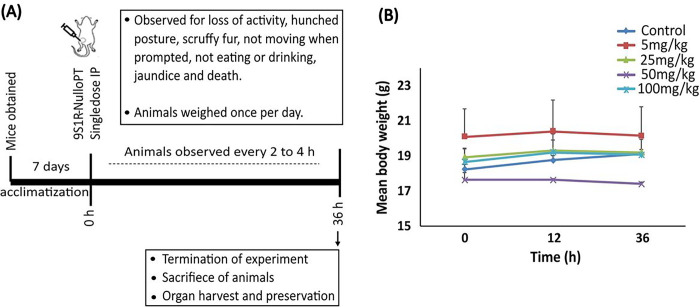
Toxicity study in mice **(A)** In vivo safety study timeline. **(B)** Changes in mean body weight plotted against time in mice treated with trehalose alone or 9S1R-NulloPT drug at 5, 25, 50 and 100 mg/kg; N=3.

To determine safety of our peptide formulation in vivo, we performed a single dose toxicity study in female BALB/c mice (Fig. 2A). The drug doses (administered I.P.) used are 5, 25, 50 and 100 mg/kg, with the control being the highest trehalose dose volume. After 36 hours of observation the experiment was terminated, and we found no significant change in the animal behavior, clinically observable signs of acute toxicity or conspicuous change in body weights among any groups (Fig. 2B). There was no significant difference between the drug and trehalose alone group. All the drug doses were well tolerated by the animals, allowing us to move forward with the preclinical in vivo cancer studies.

### Treatment with 9S1R-NulloPT inhibits tumor growth in early stage cancer but does not affect tumor volume in late stage growth.

**Figure 3:**
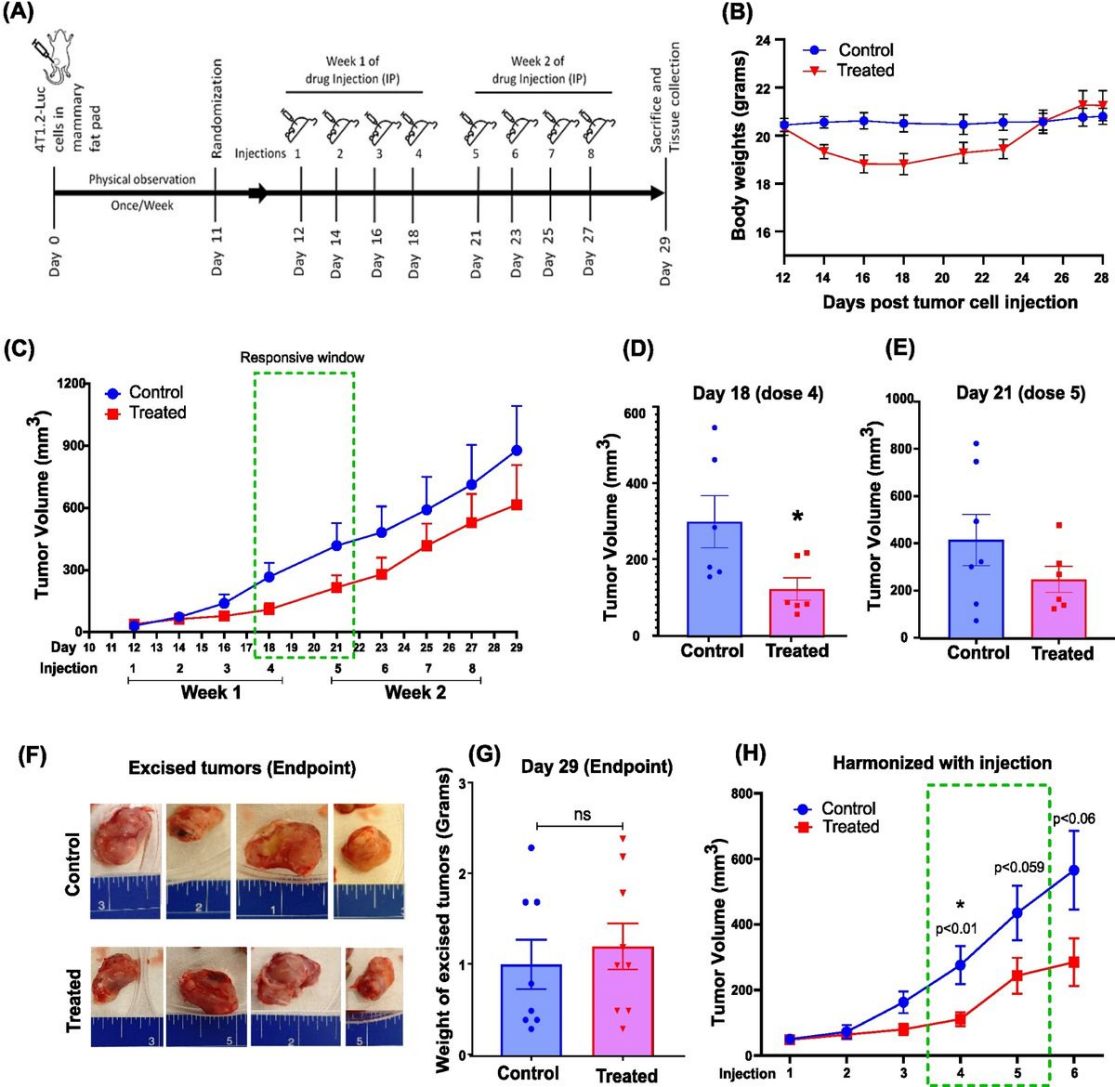
**Effect of** 9S1R-NulloPT **on tumor volume:** **(A)**Treatment schedule. **(B)** Body weight over time, in control and 9S1R-NulloPT treated mice. **(C)** Tumor volume by caliper measurement plotted against days post tumor cell injection with injection timeline of 8 doses, green box highlights drug-responsive therapeutic window with highest inter-group difference. **(D)** Cross-sectional analysis by Tumor growth [32] showing the highest responsive window on day 18, 4th dose (p<0.03, n=6–7) and **(E)** day 21, 5th dose (p<0.2, n=7). Excised tumors at endpoint from control and treatment groups; blue scale in inches. **(G)** Change in excised tumor weight from control and treatment groups. **(H)** Tumor volume measurement by harmonizing the data from both current and pilot studies, data aligned by injection timepoints. The green box depicts the responsive window with a statistically significant difference between the control and treated groups by the 4th dose (p<0.01, n=10–11). Data presented as Mean+ SEM followed by two-tailed unpaired Student’s t-test, *p<0.05 considered statistically significant.

We evaluated the effect of 9S1R-NulloPT on female BALB/c mice (8 weeks old), using a syngeneic 4T1.2-Luc orthotropic model of metastatic TNBC. We investigated the effect of the drug on tumor growth over one month in both a bilateral model (initial pilot study, **Supplementary Fig. 2**.) and a unilateral model (Fig. 3A and **Supplementary Fig. 3**). In both studies, there was a drop in weight following the first week’s treatment, however the mice later returned to near-control level weight (Fig. 3B, **Supplementary Fig. 2C**). The weight loss was never greater than 15% of the initial body weight, ruling out any adverse effects. We found that although the 9S1R-NulloPT treated mice maintained a reduced tumor volume as compared to untreated controls (Fig. 3C, **Supplementary Fig. 2B**), the decrease was statistically significant only at the fourth dose (day 18), with a highest inter-group difference (evaluated by Tumgrowth [32]) of 2.4 fold (p < 0.03), followed by the fifth dosage (day 21) with a difference of 1.7 fold (p < 0.2, not significant) (Fig. 3D, E). There was no statistically significant decrease in any other timepoints, with inter group difference at the third dosage (day 16) being 1.5 fold (p < 0.3), sixth dosage (day 23) 1.7 fold (p < 0.2), seventh dosage (day 25) 1.4 fold (p < 0.3) and eighth dosage (day 25) 1.4 fold (p < 0.4). This indicates a 9S1R-NulloPT therapy responsive window between doses 4 and 5 (Day 18–21) [32]. At the endpoint on Day 29, the size and weight of the excised tumors showed no significant difference between the treated and control groups (Fig. 3F, G, **Supplementary Fig. 3A-D**). After harmonizing (aligning the injection days), the combined results of the two studies show a significant decrease in tumor size for the treatment group, with a difference of 2.5 fold (p < 0.015, dose 4), 1.8 fold (p < 0.06, dose 5), and ~ 2 fold (p < 0.06, dose 6) (Fig. 3H) [28–31]. This suggests that the 9S1R-NulloPT drug is effective in reducing growth during the early phase of tumor development.

### 9S1R-NulloPT alters tumor metabolism but not metastasis

**Figure 4:**
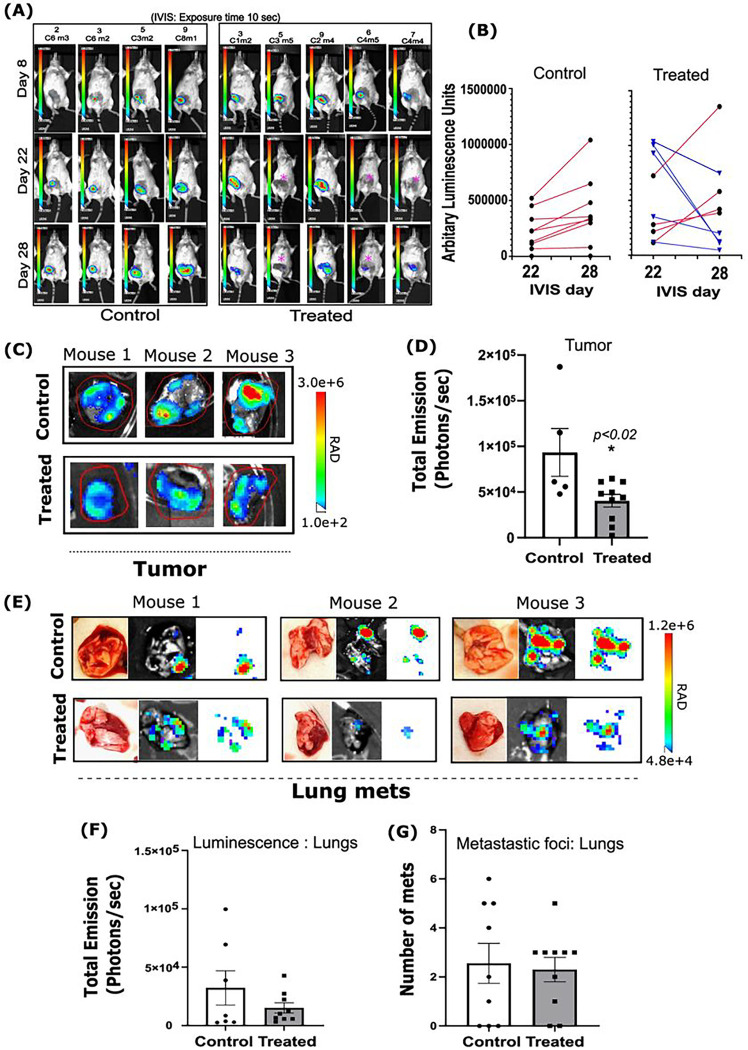
Effect of 9S1R-NulloPT treatment on tumor metabolism and lung metastasis: **(A)** Representative images taken by IVIS Spectrum (Perkin Elmer) on days 8, 22, and 28 post 4T1.2-Luc cell injection, in control and 9S1R-NulloPT treated mice, showing in vivo bioluminescence signal from the tumor cells. Pink star marks no signal in the treatment group. **(B)** Bioluminescence intensity derived from in vivo BLI at day 22 and 28 showing treated (red) and control (blue) groups with dotted lines denoting a decrease in intensity from individual mice. **(C)** Ex vivo tumor images on the same luminescent intensity scale from representative control and treated mice. **(D)** Tumors from the treated group show statistically significant (p<0.02) loss of bioluminescence represented as photons/second when compared to control tumors. **(E)** Representative photographs, images from ex vivo bioluminescent imaging (BLI) and fluorescence only signals from lungs, the secondary metastasis sites of control and treated mice. **(F)** Intensity analysis of total emission from Lungs BLIs showing a trend of reduction (not-significant, p<0.20) in bioluminescence after treatment. **(G)** Total count of metastatic foci in lungs with no significant (p<0.14) change among the groups. Data presented as Mean+ SEM followed by two-tailed unpaired Student’s t-test, *p<0.05 considered statistically significant, N=5–8.

Monitoring bioluminescence of the tumor cells in vivo revealed that, although the initial tumors (day 8) had similar signals in both control and treatment group, the control group tumors displayed increased in vivo bioluminescence with time. Five of 8 treated animals showed a reduction at the later time points (day 22 and day 28) (Fig. 4A, B). We found that the excised tumor weights among the groups were not significantly different at the end of the study (Fig. 3); however, the ex vivo BLI of the excised tumors (post mortem) showed a significant decrease in signal with treatment (Fig. 4C, D). This suggests that tumors from the treatment group have a decreased number of metabolically active 4T1.2-Luc cells. Firefly luciferase oxidizes luciferin by using ATP, Mg^2 +^ and O_2_ [33], and depletion of cellular ATP results in lower luminescence along with loss of metabolic functioning. It has been previously shown in vitro that 9S1R-NulloPT completely depletes the cellular ATP of BC cell lines within 3h and are highly lethal to hormone independent and triple negative BC cell lines (MDA-MB-231, BT-549, HS-578 T, and MDA-MB-468), as well as hormone dependent BC cell lines (MCF-7 and T-47D) [17, 18].

Although there was a trend of reduction, there was no significant change in the bioluminescence of cells metastasizing to lung (Fig. 4E, F) and no change was seen in the number of metastasis to lungs after treatment (Fig. 4G). These findings corroborated our previous study **(Supplementary Fig. 2A-I),** and support the role of 9S1R-NulloPT in altering tumor metabolism yet having no conclusive effect on metastasis [28–31].

### 9S1R-NulloPT alters tumor immune microenvironment

**Figure 5:**
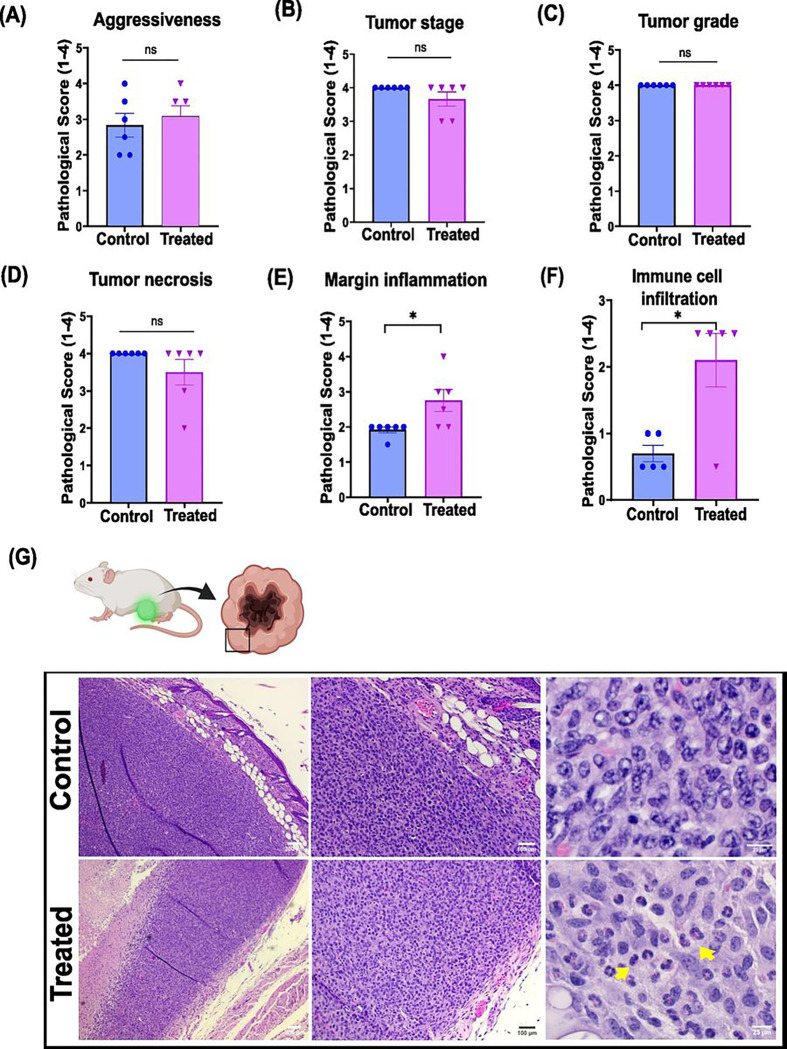
Tumor histology: Pathological scoring from the Hematoxylin and Eosin stained sections of mammary tumors from control and treated mice, revealing **(A)** aggressiveness of the tumor, **(B)** stage of tumor, **(C)** tumor grade, **(D)** score of observed necrosis, **(E)** marginal inflammation of tumor, and **(F)** infiltration of immune cells within the tumor. **(G)** Representative image from the sections at 4x (scale 200μm), 10x (scale 100μm) and 40x (scale 25μm) magnification, showing increased immune cell infiltration in the treated tumor (yellow arrows). Data represented as Mean ± SEM, Two-tailed student’s t test with * P ≤ 0.05, N=5–6.

Histopathological scoring of tumor grade and stage (on a scale of I-IV) from the H&E stained sections was performed (similar to that used for human breast tumors), per NCI’s recommendation. All the 4T1.2-Luc tumors were of very advanced stage, and had a necrotic center. Although tumors from all groups were similar in aggressiveness via grade and stage (Fig. 5A–D), there was ~ 1.5-fold increase in margin inflammation, with presence of inflammatory cells on tumor margins in the treated group. There was a 3-fold increase in the immune cell infiltration within the treated tumors compared to the control untreated group (Fig. 5E–G). The pathological report also suggests infiltration of plasma cells in the treated tumors, while none were found in the control groups. The results indicate that 9S1R-NulloPT treatment alters the TME and enhances the immune and inflammatory response. This is corroborated by the increased number of plasma cells [34], tumor-infiltrating lymphocytes (TILs) [35, 36] and higher immune scoring [37], which are all associated with a positive prognosis in TNBC patients.

### RNA seq, transcriptomics and network analysis of treated TNBC tumors

**Figure 6:**
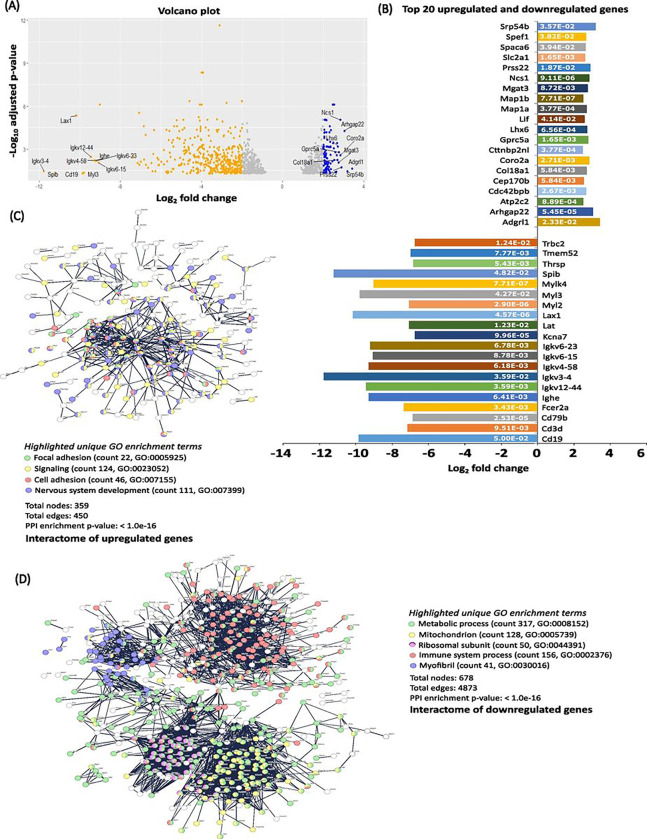
RNA sequencing and network analysis: RNA sequencing from advanced stage TNBC tumors (n=3) showing the effect of 9S1R-NulloPT treatment. **(A)** Volcano plot showing total DEGs with top 10 upregulated (orange) and downregulated (blue) genes. **(B)** Top 20 DEGs and their Log2 fold change values (X-axis) and the p-adjusted values (padj) inside the columns (white). (C-D) Interactome of the genes following STRING analysis showing DEGs involved in known and predicted protein-protein interactions with network nodes representing upregulated **(C)** and downregulated genes **(D)** Network edges (protein-protein interactions) shown in confidence view, the unique enriched GO terms are highlighted to best represent the clusters with counts and GO IDs for upregulated and downregulated interactome.

To better understand the effects of 9S1R-NulloPT treatment in advanced stage tumors, we performed post-mortem RNA sequencing followed by transcriptomics analysis. The overall gene downregulation was much higher than the level of upregulation, upon treatment, 365 genes were upregulated and 710 genes were downregulated. The top five upregulated genes (with respective Log2 fold change value) were Adgrl (3.4), Srp54b (3.2), Arghgap22 (3.0), Prss22 (2.9) and Coro2a (2.8); and the top five downregulated genes were Igkv 3–4 (−11.8), Spib (−11.2), Lax1 (−10.1), Cd19 (−9.8) and Myl3 (−9.7) (Fig. 6A–B). The function of these up- and downregulated genes are provided in **Supplementary Table 1.**

Interestingly the Igkv (immunoglobulin kappa variable) family (Igkv 12–44, Igkv 3–4, Igkv 4–58, Igkv 6–15, Igkv 6–23 and Ighe) were among the top 10 downregulated genes (Fig. 6A). We investigated the presence of clusters in the interactome network of the differentially expressed genes (DEGs) from total upregulated and downregulated genes, using STRING software (Ver 11.5) [27]. Of the upregulated DEGs, we found clusters specific to cancer, focal adhesion, signal transduction, cell adhesion and nervous system development (Fig. 6C). Network analysis of downregulated genes revealed an overall cluster for metabolic process related genes with specific clusters for mitochondria, ribosome and translation machinery, immune system and myofibril assembly (Fig. 6D). Functional enrichment analysis using the five highest significant gene ontology (GO)-terms (biological process, molecular function and cellular component) and pathways (from Kegg, Reactome and Wikipathways) of upregulated and downregulated DEGs confirmed the results [38, 39]. The details of the enrichment analysis and list of pathways are provided in Table 1.

### 9S1R-NulloPT specifically targets metabolic and bioenergetics pathways.

**Figure 7:**
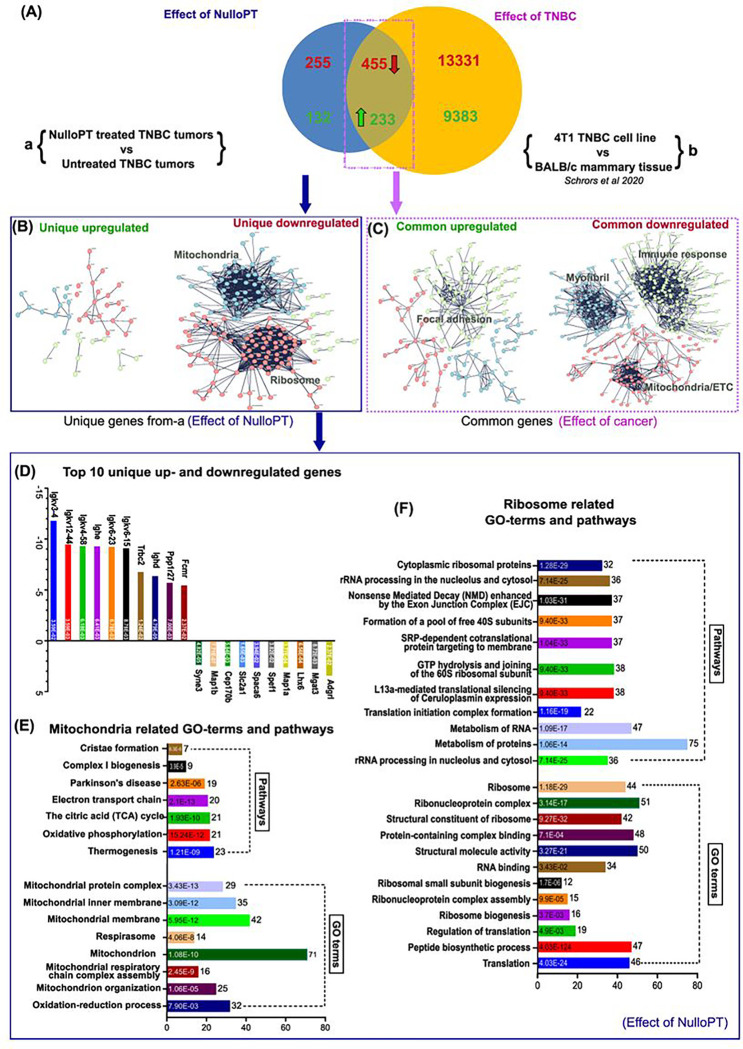
Comparative analysis of the effect of cancer vs effect of 9S1R-NulloPT treatment, in mice mammary tissue: DEGs from a published study [40] comparing 4T1.2-Luc TNBC cells originating from BALB/c mammary gland vs normal BALB/c mammary tissue (group-b) were used and compared with the DEGs obtained from the present study of 4T1.2-Luc tumors treated with 9S1R-NulloPT vs untreated tumors (group-a) to differentiate the drug-induced changes vs cancer-induced changes on the mammary tissue. **(A)** 132 upregulated and 255 downregulated genes were unique to group-a as an effect of 9S1R-NulloPT treatment and, 455 genes were commonly downregulated and 233 commonly upregulated in the two groups attributed to cancer related effects. **(B)** Cluster analysis of the unique DEGs from our study (group a) and emphasizes the drug-induced changes. No definite cluster of upregulated genes were found; however, the downregulated genes show two prominent clusters of Mitochondria related genes and Ribosome assembly/Translational machinery related genes. **(C)** Results from cluster analysis of the common DEGs emphasizing cancer induced gene expression, with focal adhesion related cluster upregulated and at least three distinct downregulated clusters comprising Mitochondrial Electron Transport Chain genes, Immune system-related genes, and Myofibril assembly genes. **(D)** Analysis of unique clusters from group-a (effect of 9S1R-NulloPT) revelated a list of top 10 up- and downregulated genes with Log2 fold change values (Y-axis) and padj scores inside the columns; a list of unique GO-terms (including biological processes, molecular function, cellular components) and pathways (including Kegg, Reactome and Wikipathways) with number of genes (X-axis) and FDR values inside columns for **(E)** Mitochondrial cluster and **(F)** Ribosomal cluster.

We found cancer related pathways, and at least 65 cancer pathway-associated DEGs, were upregulated with treatment. Since, we compared treated TNBC tumors with untreated TNBC tumors we investigated whether such cancer related pathways and DEGs are contributions of the cancer model or an effect of drug-induced change. To achieve this, we used publicly available RNA-Seq data by Schrors et al [40], comparing gene expression of 4T1.2-Luc TNBC cells (derived from BALB/c mammary gland) versus normal BAlb/C mammary tissue. This comparison identifies the DEGs that result from BC (TNBC) alone. Our 4T1.2-Luc cell line, developed by Jorcyk et al. [25], is a luciferase tagged single cell clone of 4T1.2 cells [41] with similar genomic background and tumor properties to the original 4T1, but with a higher tendency to metastasize. A comparison of the DEGs from 9S1R-NulloPT treated vs untreated tumors (group a), and 4T1 tumor cells vs mammary cells (group b) identified those DEGs that resulted from 9S1R-NulloPT treatment (unique from group a). DEGs resulting from TNBC [42, 43] are common to group a and b (Fig. 7).

The comparison between the RNA-Seq results revealed that 688 DEGs were due to TNBC (common to group a and b), with 233 genes upregulated and 455 genes downregulated (Fig. 7A). Cluster analysis of these genes using STRING (Ver 11.5) revealed the TNBC downregulated clusters consist of myofibril assembly, immune related and mitochondrial electron transport chain (ETC)TC related genes. The TNBC upregulated cluster comprises focal adhesion and cancer signaling related genes (Fig. 7C). Although no noteworthy cluster was found in upregulated genes unique to 9S1R-NulloPT treated cancer, Abl-1 and Shc-1 formed the center node of a small cluster. Remarkably, when analyzed in details **(Supplementary Fig. 5),** among the above-mentioned cancer related pathways we found only 20 associated genes to be uniquely upregulated in 9S1R-NulloPT treated tumors **(Supplementary Fig. 5A-C)** including pan-cancer related genes such as Shc-1 [44], Mtor [45], Lrp6 and Wnt5a [46]. Interestingly, a recent report suggests that Wnt5a [47] and Abl-1 [48] are potent suppressors of TNBC progression and are associated with a better prognosis in BC. We also found 44 DEGs associated with at least 12 cancer pathways that were upregulated in both 9S1R-NulloPT treated and TNBC untreated tumors **(Supplementary Fig. 5, D-F),** signifying a contribution from the TNBC model. A list of cancer pathway related DEGs in TNBC untreated tumors and 9S1R-NulloPT treated tumors is provided in **Supplementary Fig. 6**.

The unique downregulated clusters, which signify the effect of 9S1R-NulloPT only (and not breast cancer), comprise mitochondrial ATP synthesis coupled proton transport (Atp5e, Atp5j, Atp5k, Atp5h and Atp5j2), mitochondrial electron transport chain (Uqcrb, Uqcrh, Ndufv2 and Ndufs6), Oxidative phosphorylation (Cox6c, Atp5e, Uqcrb, Ndufs6, Atp5j, Cox7a2, Atp5k, Uqcrh, Atp5h, Ndufv2 and Atp5j2), Mitochondrial respiratory chain complex-1(Ndufv2, Ndufs6, Ndufs5, Ndufa5, Ndufb5, Ndufs4 and Ndufb3) and ribosome/translation related genes (Rpl17, Rpl19, Rps27l, Rpl22l1, Rpl9, Rps14, and Rps27) (Fig. 7B, E, F). The top 5 unique downregulated genes were from the Igkv family (Igkv 3–4, Igkv-12–44, Igkv 4058, Ighe and Igkv6–23) and top 5 unique upregulated genes were Adgrl, Mgat3, Lhx6, Map1a and Spef1 (Fig. 7D). Enrichment analysis of the mitochondria cluster revealed Cristae formation, Complex I biogenesis, Oxidative phosphorylation, Electron transport chain, TCA cycle pathways and the GO-terms such as respirasome, mitochondrial respiratory chain assembly and mitochondrion organization (Fig. 7E). Enrichment analysis of the ribosomal cluster revealed Ribonucleoprotein complex, RNA binding, Ribosomal biogenesis, Translation and Peptide biosynthesis pathways and the GO-terms such as rRNA processing, SRP dependent cotranslational protein targeting to membrane, L13a mediated silencing of Ceruloplasmin expression (Fig. 7F). Interestingly, low ceruloplasmin expression correlates with a favorable prognosis and tumor immune cell infiltration in BC patients [49]. We found 149 genes which were upregulated with TNBC (in group-b) were downregulated as an effect of 9S1R-NulloPT treatment (in group-a). These genes comprise the same cluster of Ribosome and Mitochondria genes with similar GO-terms and pathways **(Supplementary Fig. 6A-C)**. Likewise, 99 genes which were downregulated as an effect of TNBC (group-b) were upregulated due to 9S1R-NulloPT treatment (group-a), and these includes Focal adhesion, nervous system development, ECM organization related genes **(Supplementary Fig. 6D-F)**. In summary, the RNAseq suggest that 9S1R-NulloPT targets metabolic pathway related genes, specifically the Mitochondrial energy metabolism and Ribosome associated pathways such as Ribosome biogenesis and translation.

## Discussion

This is the first study to highlight the physiological impact nullomer-based peptide drugs delivered in vivo. 9S1R-NulloPT (9S1R peptide in trehalose) was very well tolerated in vivo when delivered intraperitoneally in mice. Our in vivo RNAseq results support previous work showing that 9S1R-NulloPT targets metabolic pathways, specifically the genes for mitochondrial and ribosomal functions. The effect of this drug on mitochondrial physiology and ATP production has been demonstrated in cancer cell lines [17, 18] where it is reported that 9S1R-NulloPT reduces ATP formation and mitochondrial membrane potential, and increases mitochondrial reactive oxygen species (ROS) generation. A moderate to mild mitochondrial stress and fission is beneficial for TNBC cell growth and aggressiveness, but severe mitochondrial stress and increased fission leads to excessive ROS generation and apoptosis of cancer cells [50]. The drastic alteration in the mitochondrial pathways in the 9S1R-NulloPT treated tumors shows that the peptides target mitochondria directly or indirectly, which could be exploited as a potential mitochondria-targeted anti-cancer/tumor therapy. Ribosome synthesis in the nucleolus increases in cancer cells to cope with increased demand for protein synthesis. Ribosome biogenesis-targeting (CX-3543, CX-5461 [51, 52] and BMH [53]) is still in its infancy, however, it is emerging as an effective cancer therapy used in multiple clinical trials [54]. Biogenesis of ribosomes make an interesting target for cancer chemotherapy for many reasons: (1) the inhibition of ribosome biogenesis induces cell cycle arrest in a p53-independent manner [54], (2) these inhibitions don’t affect the resting cells, possibly due to the long half-life of cytoplasmic ribosomes [55], and (3) it could lead to apoptosis of neoplastic cells that have a high nucleolar ribosomal biogenesis rate [56]. Ribosome synthesis is extremely complex and one of the most energetically demanding cellular activities [57]. We hypothesize that our drug 9S1R-NulloPT targets mitochondrial ATP production and ribosome biogenesis leading to a loss of metabolic activity of the cells. To ensure more efficient killing of these aggressive TNBC cells in vivo and to enable tumor size reduction, we plan to use our drug synergistically along with other anticancer drugs that act through different cytotoxic pathways [58–61].

RNAseq analysis revealed upregulation of: unique clusters of ECM organization genes (collagen formation and degradation genes such as Col6a3, Col5a3 Plod1, Mmp3, Loxl3 and Plec); focal adhesion genes (Itga5, Lamc1, shc1, Arghap35, and Pxn), and cytoskeletal protein binding genes (Abl1, Map1a, Map1b, Map6, Myo10 and Trak1). Among the highest upregulated genes Lhx6 was noteworthy in relevance to BC, as it is reported to suppress activation of the PI3K/Akt/mTOR signaling, inhibiting the progression of BC [62]. The highest downregulated genes belonged to the Igkv family, which has been suggested as an identifying biomarker for TNBC cancers [63]; and the Fcmr gene whose knock down leads to increased phagocytosis, enhanced antigen presentation, and heightened T cell activation. Fcmr is also a promising anti-cancer target [64].

Although treatment did not change tumor size post necropsy, the in vivo as well as ex vivo BLI of the tumors showed reduced bioluminescence in the treated group. There were multiple mice (~ 50%) in the treatment group that showed a reduction in bioluminescence which implies less cells being present in the tumor due to decreased proliferation or an increased cell death and loss of metabolic activity of the tumor. This corroborates the results obtained in the pilot study. Ex vivo BLI of the tumors in both the studies showed a significant decrease in bioluminescence and thus a decrease in the metabolic activity of the tumor cells in the treated groups but not in controls. This suggests that although the treatment does not change the size of tumors drastically, it altered the metabolism of the tumor cells rendering them inactive. This makes sense in light of the tumor transcriptomics: metabolism related genes were mostly downregulated in the treated tumors, specifically the mitochondrial and ribosomal genes that are essential for energy production and metabolism. The in vivo BLI from day 22 (24h after dose 5) clearly shows the reduced bioluminescence in treated tumors during this period. For the pilot study (using a bilateral tumor model) we imaged mice after the 4th 9S1R-NulloPT dose (day 22, **Supplementary Fig. 4A**) and found a similar decrease in in vivo bioluminescence in the treated groups.

Histopathological evaluation confirmed that treatment and control tumors had the same grade, stage and aggressiveness. However, it was interesting to find that there was a significant change in the tumor immune-microenvironment of the treated tumors. Immune cell infiltration increased significantly in the 9S1R-NulloPT treated tumors, as measured by plasma cell numbers and margin inflammation. A positive correlation of plasma cells with favorable patient outcomes has recently been reported, [34] suggesting a better prognosis. It is well established that the outcome of immunotherapy treatment, and thus prognosis of BC, is dictated by the tumor microenvironment. Recent reports suggest that the immune score of BC patients could be useful for treatment decisions and survival prediction [37]: activated immune cell infiltration in tumors correlates with better prognosis [35]. Increased levels of tumor infiltrating lymphocytes (TILs) have been associated with disease-free status and overall survival rates in TNBC patients with and without any treatment. The presence of TILs in the breast tumor microenvironment can also predict responses to neoadjuvant therapy and adjuvant chemotherapy treatments, and high numbers of TILs correlate with increased pathological complete responses in TNBC [36]. We also found that the 9S1R-NulloPT treatment resulted in less externally visible necrosis as compared to untreated tumors or trehalose treated groups (data not shown). There was no conspicuous difference between the PBS control and trehalose groups **(Supplementary Fig. 4A-G),** suggesting that the immunological changes in the tumor microenvironment (TME) are a specific contribution of the nullomer peptide.

The results of this study should be seen in the light of the fact that the peptides were not stabilized and have a half-life in serum of about 9 minutes. Small peptides such as 9S1R tend to have shorter half-life in circulation, but when internalized their response could be effective immediately as we have seen in MDA-MB-231 cell lines [18]. Cellular internalization studies **(Supplementary Fig. 1)** show that the peptide could be identified from crude lysed cytosol fraction (cytosol + mitochondria fraction) in several valence forms (2+, 3+, 4+) from m/z 380.7 0.1 when incubated with 4T1.2 cells for at least two hours. This confirms that the 9S1R-NulloPT are internalized within the cells and are sequestered into other forms, which are more stable than those in serum. Biochemical properties of 9S1R are similar to known mitochondria penetrating peptides in charge, hydrophobicity, poly arginine content [65], and the effects on mitochondrial functions[17]. All of this suggests mitochondrial localization. However, the mechanism of the peptide’s metabolism, uptake and its sub-cellular localization warrants further investigation.

The Nullomer peptides are readily solubilized in the sugar trehalose, which has known anti-cancer properties [23, 66]. We used the nullomer-trehalose combination with the peptoprime 9R- and its scrambled version 9S1R to screen the NCI 60 cancer panel and the TNBC model MDA-MB-231 and found them to be effective in vitro. Another peptoprime linked to 5 arginines, did not show anticancer effects, and is used as a negative control peptoprime [17, 18]. As noted above, trehalose is reported to exhibit anticancer properties, and in this study, we did see some effects **(Supplementary Fig. 2–5).** Trehalose alone had similar potency in tumor size reduction as 9S1R-NulloPT groups in study-2 **(Supplementary Fig. 3),** but a contrasting higher than control tumor size in study-1 **(Supplementary Fig. 2),** with no significant change in post mortem tumor size. Similar to the control group, Trehalose did not show any effect on the immune cell infiltration or margin inflammation on the tumors **(Supplementary Fig. 4).**

The 4T1.2-Luc cells have a specific tendency to metastasize into lungs and bone. We found specific metastasis to the lungs, and although there was a trend of reduced bioluminescence in the treated lungs as compared to controls, it was not statistically significant, this is corroborated in the pilot study. There was also no significant change in metastatic foci counts in the lungs. These metastatic findings are still preliminary, primarily because of the large variations in the control groups.

Overall, we show a moderate level of therapeutic potential of 9S1R-NulloPT, in terms of tumor growth. In both of our in vivo studies, the treatment decreased the tumor volume moderately with statistical significance during the growth phase of the tumor (day 18) by the fourth dose, however in the end the control and treatment group had similar tumor weights. This suggests that 9S1R-NulloPT acts during an early therapeutic window in this TNBC model.

### Caveats in the study and future plans:

1. The efficacy of the peptide in reducing tumor volume wasn’t as large as the metabolic inhibition and this could due to (a) the short half-life of the peptide in serum, (b) the dramatic rate of proliferation of TNBC cells that may be greater than rate of clearance of the cells from the tumor, making them necrotic and inactive but still large in size. To circumvent this in the future we plan to (a) enhance the peptide stability by loading the drug in an LNP or extracellular vesicle carrier (b) resect the tumor after dose 5 and continue drug administration to evaluate the tumor signatures and metastasis by in vivo BLI.
2. The RNA sequencing results revealed mitochondrial and ribosomal genes to be greatly affected by 9S1R-NulloPT treatment, but this needs to be confirmed by PCR. We also plan to identify the targets directly by the subcellular localization of the 9S1R-NulloPT.
3. To improve the therapeutic effect of the drug we plan to combine it with a known chemotherapy drug such as Doxorubicin along with 9S1R-NulloPT in in vivo TNBC models.
4. RNA seq analysis should be repeated with mammary tissue from normal mice, those with TNBC tumors, and those with TNBC tumors treated with 9S1R-NulloPT, in at least two different stages (day 18 and day 27).
5. We did not perform any intravenous administration as we found previously that 9S1R-NulloPT has a low but significant hemolytic activity of 0.5% at 10 μM concentration on RBCs [17].

## Conclusion

**Figure 8:**
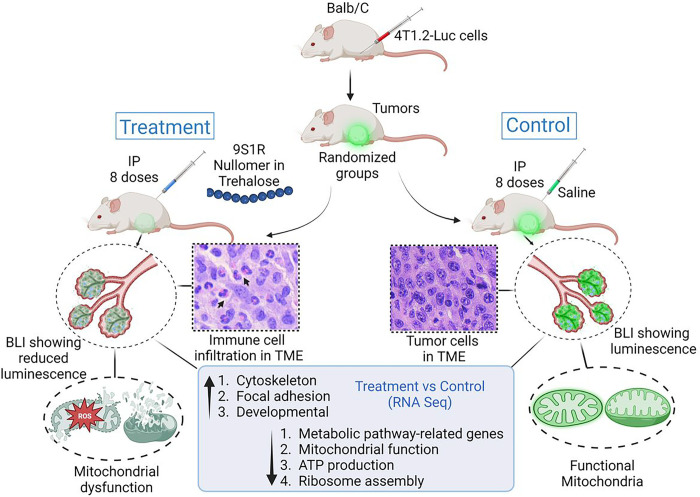
Schematic representation of the study and major findings. Image made with Biorender.

Previously in vitro experiments with the NulloPTs showed very promising results using the NCI 60 panel of cells. he current in vivo mouse study (Fig 8) demonstrates that the drug reduces early tumor volume (up to the 5th treatment) with no significant change in terminal tumor mass. The treatment modifies the tumor microenvironment by rendering it metabolically inactive, as shown by the reduced bioluminescence from the excised tumor and the downregulation of metabolic pathway-related genes involved with mitochondrial function, ATP production, and ribosome assembly. These results corroborate our previous findings from the NCI 60 panel, where we have shown that the 9S1R-NulloPT leads to a drastic decrease in mitochondrial membrane potential and ATP generation, and a noticeable increase in mitochondrial ROS generation.

## Figures and Tables

**Table 1:** Functional enrichment analysis from STRING interactome: List of top five **(A)** GO terms and **(B)** pathways, based on significance of enrichment or false discovery rate (FDR) shown as a measure of p-values corrected using Benjamini-Hochberg procedure for the upregulated and downregulated DEGs.

(A) Functional enrichments in network: Top 5 GO terms based on significance FDR					
Upregulated genes					
	GO-term	Description	Count in network	Strength	FDR
**Biological process** **(Gene ontology)**	GO:0009653	Anatomical structure morphogenesis	117 of 2244	0.51	3.97E-26
	GO:0032502	Developmental process	192 of 5629	0.32	3.73E-25
	GO:0048856	Anatomical structure development	184 of 5258	0.33	4.96E-25
	GO:0007275	Multicellular organism development	176 of 4921	0.34	1.35E-24
	GO:0065007	Biological regulation	275 of 10591	0.2	1.35E-24
**Molecular function** **(Gene ontology)**	GO:0005515	Protein binding	204 of 6764	0.27	9.56E-21
	GO:0005488	Binding	272 of 11199	0.17	8.13E-19
	GO:0008092	Cytoskeletal protein binding	57 of 947	0.57	7.5E-14
	GO:0003779	Actin binding	35 of 418	0.71	1.3E-11
	GO:0044877	Protein-containing complex binding	65 of 1411	0.45	4.98E-11
**Cellular component** **Gene ontology)**	GO:0030054	Cell junction	96 of 2050	0.46	5.05E-18
	GO:0110165	Cellular anatomical entity	328 of 15632	0.11	5.05E-18
	GO:0005856	Cytoskeleton	94 of 2060	0.45	3.84E-17
	GO:0005622	Intracellular	286 of 12596	0.14	1.35E-16
	GO:0015629	Actin cytoskeleton	41 of 458	0.74	2.95E-15
Downregulated genes					
**Biological process** **(Gene ontology)**	GO:0006955	Immune response	115 of 979	0.58	6.93E-29
	GO:0002376	Immune system process	156 of 1842	0.44	4.17E-26
	GO:0051707	Response to other organism	114 of 1145	0.51	2.77E-23
	GO:0044419	Interspecies interaction between organisms	118 of 1309	0.47	4.23E-21
	GO:0006412	Translation	56 of 316	0.76	1.98E-20
**Molecular function** **(Gene ontology)**	GO:0003735	Structural constituent of ribosome	52 of 155	1.04	9.37E-30
	GO:0005198	Structural molecule activity	63 of 483	0.63	4.58E-17
	GO:0015078	Proton transmembrane transporter activity	20 of 113	0.76	0.00000298
	GO:0046933	Proton-transporting ATP synthase activity, rotational mechanism	8 of 16	1.21	0.00019
	GO:0003785	Actin monomer binding	9 of 24	1.09	0.00019
**Cellular component** **(Gene ontology)**	GO:0110165	Cellular anatomical entity	607 of 15632	0.1	6.84E-28
	GO:0005737	Cytoplasm	467 of 10283	0.17	7.46E-28
	GO:0005840	Ribosome	55 of 213	0.92	1.68E-27
	GO:0044391	Ribosomal subunit	50 of 181	0.95	4.18E-26
	GO:0022626	Cytosolic ribosome	40 of 99	1.12	8.46E-26
**(B) Functional enrichments in network: Top 5 pathways based on significance FDR**					
Upregulated genes in pathway					
	**GO-term**	**Description**	**Count in network**	**Strength**	**FDR**
**(A) Functional enrichments in network: Top 5 GO terms based on significance FDR**					
**Biological process** **(Gene ontology)**	mmu04510	Focal adhesion	18 of 196	0.75	0.00000403
	mmu04360	Axon guidance	16 of 176	0.75	0.000015
	mmu04512	ECM-receptor interaction	10 of 87	0.85	0.0004
	mmu04810	Regulation of actin cytoskeleton	15 of 212	0.64	0.0004
	mmu05205	Proteoglycans in cancer	13 of 199	0.6	0.0027
**Molecular function** **(Gene ontology)**	MMU-1474244	Extracellular matrix organization	25 of 295	0.72	0.000000133
	MMU-162582	Signal Transduction	79 of 2417	0.3	0.00000163
	MMU-422475	Axon guidance	22 of 278	0.69	0.00000202
	MMU-9006934	Signaling by Receptor Tyrosine Kinases	25 of 418	0.57	0.0000199
	MMU-194315	Signaling by Rho GTPases	22 of 381	0.55	0.00018
**Cellular component** **Gene ontology)**	WP488	Alpha 6 beta 4 integrin signaling pathway	12 of 66	1.05	0.000000793
	WP85	Focal adhesion	16 of 183	0.73	0.0000146
	WP2573	Primary focal segmental glomerulosclerosis (FSGS)	10 of 72	0.93	0.0000499
	WP65	Insulin signaling	14 of 158	0.74	0.0000499
	WP6	Integrin-mediated cell adhesion	11 of 99	0.83	0.0000629
Downregulated genes in pathway					
**Biological process** **(Gene ontology)**	MMU-1799339	SRP-dependent cotranslational protein targeting to membrane	40 of 88	1.17	1.11E-26
	MMU-975956	Nonsense Mediated Decay (NMD) independent of the Exon Junction Complex (EJC)	39 of 90	1.15	1.08E-25
	MMU-72689	Formation of a pool of free 40S subunits	40 of 97	1.13	1.08E-25
	MMU-156827	L13a-mediated translational silencing of Ceruloplasmin expression	41 of 107	1.1	1.08E-25
	MMU-72706	GTP hydrolysis and joining of the 60S ribosomal subunit	41 of 108	1.09	1.08E-25
**Molecular function** **(Gene ontology)**	MMU-1799339	SRP-dependent cotranslational protein targeting to membrane	40 of 88	1.17	1.11E-26
	MMU-975956	Nonsense Mediated Decay (NMD) independent of the Exon Junction Complex (EJC)	39 of 90	1.15	1.08E-25
	MMU-72689	Formation of a pool of free 40S subunits	40 of 97	1.13	1.08E-25
	MMU-156827	L13a-mediated translational silencing of Ceruloplasmin expression	41 of 107	1.1	1.08E-25
	MMU-72706	GTP hydrolysis and joining of the 60S ribosomal subunit	41 of 108	1.09	1.08E-25
**Cellular component** **(Gene ontology)**	WP163	Cytoplasmic ribosomal proteins	35 of 77	1.17	5.24E-24
	WP295	Electron transport chain	36 of 97	1.08	1.34E-22
	WP1248	Oxidative phosphorylation	26 of 58	1.16	6.23E-18
	WP1253	Type II interferon signaling (IFNG)	10 of 32	1.01	0.0000161
	WP2271	Macrophage markers	5 of 10	1.21	0.0022

## Data Availability

The datasets used and/or analyzed during the current study are available from the corresponding author on reasonable request.

## List of abbreviations

9S1R NulloPT: 9S1R nullomer peptide in trehalose
aa: amino acid
BC: Breast cancer
BLI: Bioluminescence imaging
DEGs: Differentially expressed genes
ECM: Extracellular matrix
EIC: Extracted ion chromatogram
ER: Estrogen receptor alpha
ETC: Electron transport chain
FDR: False discovery rate
GO: Gene ontology
H&E: Hematoxylin and Eosin
HER: Human epidermal growth factor receptor
IP: Intraperitoneal
IVIS: In vivo imaging system
MS: Mass Spectrometry
mTNBC: metastatic TNBC
MTP: Mitochondria penetrating peptide
NulloP: Nullomer Peptide
NulloPT: Nullomer peptide in trehalose
Padj: P adjusted values
PD 1: Programmed cell death protein-1
PDL1: Programmed cell death Ligand-1
PR: Progesterone receptors
ROS: Reactive Oxygen Species
SEER: Surveillance, Epidemiology, and End Results
TCA: Tri Carboxylic Acid Cycle
TIL: Tumor infiltrating lymphocyte
TME: Tumor microenvironment
TNBC: Triple Negative Breast Cancer
